# RETRACTION: Evaluation of Genetic Variations in Maize Seedlings Exposed to Electric Field Based on Protein and DNA Markers

**DOI:** 10.1155/bmri/9878701

**Published:** 2026-04-03

**Authors:** BioMed Research International

RETRACTION: A. A. AL‐Huqail and E. Abdelhaliem, “Evaluation of Genetic Variations in Maize Seedlings Exposed to Electric Field Based on Protein and DNA Markers,” *BioMed Research International* 2015, (2015): 874906; https://doi.org/10.1155/2015/874906.

The above article, published online on 24 June 2015 in Wiley Online Library (http://wileyonlinelibrary.com), has been retracted by agreement between the Editorial Board and John Wiley & Sons Ltd. A third party reported that images of comet assays in Figure 4 showed evidence of manipulation. An investigation by the publisher found evidence of image manipulation in Figure 4 as well as evidence of duplicated bands in Figure 1, Figure 2, and Figure 3. The authors did not respond to an inquiry and request for original data by the publisher.

The retraction has been agreed to because the evidence of image manipulation fundamentally compromises the results presented in the article.

